# Dr. Allen Augustus Stevens

**Published:** 1893-11

**Authors:** 


DR. ALLEN AUGUSTUS STEVENS, of Sinclairville, Chautauqua county, N. Y., died at his residence on Friday, October 13, 1893, aged forty-five years. Dr. Stevens was a graduate of the Buffalo University Medical College, in the class of 1873, and immediately began to practise medicine in his native town, Sinclairville, and so continued for twenty years.

He married, soon afterward, Mary, daughter of Hon. Orsamus A. White, of Norwalk, O. His widow, with three children, survive; and he also leaves behind an aged father and mother, together with two sisters.

Dr. Stevens was respected as a citizen, and was largely interested in whatever advanced the interests of the community; especially was he active in promoting educational interests. As a physician he was accomplished and skilful, as well as eminent and popular. He was a member of the Medical Society of the County of Chautauqua. The funeral was largely attended at his late home on Park avenue, conducted by the Masonic fraternity, in charge of F. A. Shaw, assisted by Rev. Dr. Rafter, of Dunkirk. Five orders, or societies, in full regalia were present, and the services throughout were imposing and impressive. The bearers were Doctors E. M. Scofield, E. A. Scofield, Vosburg, Tompkins, Sales, and Harrison. The interment was in Evergreen cemetery.

